# Physico-Mechanical Properties and Hydration Processes of Cement Pastes Modified with Pumice, Trass and Waste Chalcedonite Powder

**DOI:** 10.3390/ma17010236

**Published:** 2023-12-31

**Authors:** Edyta Spychał, Martin Vyšvařil

**Affiliations:** 1 Department of Technology and Organization of Construction, Faculty of Civil Engineering and Architecture, Kielce University of Technology, Al. Tysiąclecia Państwa Polskiego 7, 25-314 Kielce, Poland; 2 Institute of Chemistry, Faculty of Civil Engineering, Brno University of Technology, Veveří 331/95, 602 00 Brno, Czech Republic; vysvaril.m@fce.vutbr.cz

**Keywords:** cement paste, reduce CO_2_, additive, pumice, trass, waste chalcedonite powder, consistency, rheological measurements, compressive strength, hydration process

## Abstract

In this article, the physico-mechanical properties and hydration processes of cement pastes containing three additives are introduced. Cement was replaced with pumice, trass, waste chalcedonite powder at 30% by mass and a combination of pumice or trass and waste chalcedonite powder in the amounts of 15% each. The main aim of this research was to assess the properties of two- or three-component binders to save cement in these binders. Rheological properties such as consistency, yield stress, viscosity and thixotropy were determined, in addition to porosity, 7-day and 28-day flexural and compressive strength and bulk density. Additionally, the heat evolution and degree of hydration of the tested pastes were compared. The use of all additives resulted in a reduction in the consistency of the tested pastes. The highest compressive strength measured after 28 days was observed for the cement paste with a 30% content of waste chalcedonite powder, which is related to it having the best pozzolanic activity of the materials used. The results of this research have confirmed that pumice, trass and waste chalcedonite powder can be used as components of blended Portland cements.

## 1. Introduction

Cement is a basic binder used in the production of diverse pastes, mortars and concretes. The cement industry is characterized by high emissions and high energy and material consumption. It is estimated that the production of 1 ton of Portland clinker causes emissions of about 0.8–0.9 tons of CO_2_ [1,2]. Cement production is responsible for about 5% of global carbon dioxide emissions [3,4]. Greenhouse gas emissions such as NO_x_, SO_2_, CO and CO_2_ are a serious problem worldwide [1,3,4,5,6]. Different actions are constantly taken to limit this phenomenon. The most important suggestions for solutions to reduce carbon dioxide emissions in the cement industry worldwide are [1,2,3,4,5,6,7,8,9,10,11,12]:

- The replacement of Portland clinker with other materials (pozzolanic and waste materials), resulting in a new type of Portland cement with additions—compositions with two– or three–ingredient binders;
- The popularization of multi-component and low–clinker cements;
- The efficient use of raw materials and alternative fuels (e.g., biomass);
- The popularization of energy-saving technologies (e.g., belite, sulfoaluminate).

Significant topics in cement production include not only the reduction of environmental pollution or reduction of emissions of CO_2_ but also the protection of the natural environment through the use of waste materials [1,2,4,13,14,15]. Siliceous fly ash, granulated blast furnace slag and limestone powder are the most popular non-clinker raw materials [1,5]. Due to the limited quantity of siliceous fly ash and granulated blast furnace slag in the decarbonization of the energy and metallurgy sectors, other alternative materials are being looked for [1,9]. The best solutions include raw materials with pozzolanic properties that do not require additional thermal or chemical treatment, such as zeolite, spongilite, diatomite, pumice and trass [16,17,18,19,20,21]. Some of these natural pozzolans have been used to improve the performance of non-hydraulic binders since ancient times. Pozzolanic materials can have a positive impact on the durability of building materials, especially on their rheological and mechanical properties. These effects are related to the amelioration of the pore structure caused by the calcium silicate hydrate (C–S–H) phase, which is an effect of a pozzolanic reaction [22]. Pumice is a raw material produced from solidified igneous volcanic rock lava. The cellular structure of pumice is the result of the formation of bubbles or air voids when gases contained in lava flowing from volcanoes are trapped during cooling [23,24]. Studies in which cement was partially replaced in pastes were carried out by, among others, the authors of the publications [19,21,23]. Kocak et al. [19] tested cement mortars modified with pumice and diatomite, and they replaced 5% or 10% of the cement with the addition of one of these or 10 or 20% with their combination. The authors stated that pumice and diatomite were characterized by lower pozzolanic activity at the early stages and a higher activity at the later stages. Cement mortars with 10% and 20% contents of one of the additions, tested after 28 days, met the requirements for cement class 42.5. Hossain [23], in his research, replaced cement with a content of pumice from 0% to 25%. The pumice caused a delay in the initial and final setting times of the pastes by about 15% compared to cement paste without any pumice addition. This raw material caused a decrease in the compressive strength of more than 25% when cement was replaced with a 25% content of pumice. Hamade et al. [25] conducted a comprehensive review of the effects of pumice on the mechanical properties of cement concrete. In most of the analyzed research, an increase in the amount of pumice leads to a lower apparent density, a reduction in workability and a reduction in mechanical properties. Trass is a material of volcanic origin and comes from pyroclastic rocks [26]. The analysis of the influence of trass on the properties of cement composites has not been as widely considered as the case of pumice. Kapeluszna et al. [17] tested cement pastes and mortars modified with 10% and 25% trass. This had a slight impact on the water demand of the cements. As it turned out, an increase in this material resulted in a shortening of the setting time of the pastes, reducing the consistency of the mortars and reducing their flexural and compressive strengths. On the other hand, the results of the compressive strength testing of mortars with 25% content of trass after 28 days were higher than the results of the 7-day strength testing, which proves the pozzolanic properties of this material.

A good idea in the production of Portland cement with additions and low-clinker cements is the use of waste stone powders such as granite, marble, basalt or chalcedonite powder [15,27,28,29,30,31,32,33,34]. Abdelaziz M.A. et al. [29] tested cement pastes and mortars modified with limestone dust and basalt dust. In their opinion, it is beneficial to use 4–12% content by mass of quarry dust—the mechanical properties of these modified mortars were better than those of the control mortars. Prokopski et al. [30] show that granite powder in dry cement mortars can increase workability. Stone waste in the amount of 50–100 kg/m^3^ can increase compressive strength by about 15–40%. The greater the amount of granite powder, the higher the adhesion of mortars. The positive influence of granite powder (in the amounts of 5% and 10% by mass) on consistency and water absorption was confirmed by Woźniak et al. [32]. The replacement of cement with an optimum 10% content of granite powder reduced the mechanical properties of masonry mortars. According to the authors, the optimal amount of this stone powder is 10% by mass of cement if we want to achieve an acceptable reduction in bending and compressive strength. Chalcedonite powder is produced as a waste product during the production of aggregates from rocks of sedimentary origin [35,36]. Its use as a substitute for cement composites is not widely known. Only a few publications are available on this subject (similar to trass). Naziemiec et al. [37] presented examples of the use of chalcedonite in building and road engineering, the cement industry and sanitary engineering in a general way. Kotwa et al. [38] tested concretes with a constant amount of waste chalcedonite powder, which was equal to 15% relative to the cement. The addition of stone powder caused a reduction in compressive strength by a maximum of about a few percent compared to the results for strength for different classes of concrete. Water absorption for modified concretes was obtained in the range from 4.2% to 4.9%. In publication [15], cement mortars were modified with chalcedonite powder in amounts of 5%, 20%, 35% and 50%. Using this material in the range from 5% to 35% reduced compressive strength by a maximum of about 31% after 28 days and by a maximum of about 38% compared to reference concretes. Samples with 5% and 20% chalcedonite powder were characterized by the best mechanical properties.

In the literature, there is research in which two– or three–component binders are used [5,39,40,41,42,43,44]. This solution enables the use of the positive properties of individual raw materials. Often, the synergistic effect of two or three ingredients results in comparable or even better mechanical properties than the paste or mortar without modification or material with only one additive [5,43,44]. 

The purpose of the research in this article includes the possibility of reducing carbon dioxide emissions in the cement industry by replacing a part of cement with one or two other ingredients. The focus of the research was two- or three-component binders. By analyzing the available literature and the possibilities of using various amounts and types of raw materials other than cement, two natural additives were selected for research (pumice and trass) as well as waste natural material from aggregate production—waste chalcedonite powder. The research program included replacing cement with 30% additive(s) by mass and obtaining the composition of CEM II cement—Portland cement B variety (almost the upper limit for the amount of additive). The purpose of the additive combinations used was to assess the most favorable cement–additive(s) system among the selected ones. It was important not only to assess the properties of pastes modified with one of the additions but also to assess their interaction in the three-component binder, due to the possibility of using waste materials. The research mainly focuses on the effects of these additives on the physical and mechanical properties of blended cement pastes. Consistency research was supplemented by rheological measurements, especially the determination of yield stress and viscosity. Additionally, the paper analyses the role of pumice, trass and waste chalcedonite powder in hydration processes. 

## 2. Materials and Methods

### 2.1. Materials 

In this research, commercial cement CEM I 42.5 R (Górażdże Cement S.A., Górażdże, Poland); three additives, pumice (Vulkalit WR, Vulcatec Riebensahm GmbH, Kretz, Germany), trass (Tubag, Sievert Poland Sp. z o.o., Strzelin, Poland) and waste chalcedonite powder (Crusil Sp. z o.o., Inowłódz, Poland); and tap water were used. All additives were used in the form of fine powder. The chemical composition of the cement and additives is introduced in Table 1. This was measured with an Axios X-ray fluorescence (XRF) spectrometer (Malvern Panalytical Ltd., Royston, UK). It is evident that pumice, trass and waste chalcedonite powder containing more than 70% hydraulic oxides (SiO_2_, Al_2_O_3_, Fe_2_O_3_) represented a very good prerequisite for the pozzolanic activity of these materials and met the requirements for N and F pozzolans according to ASTM C618 [45].

**Figure 1:**
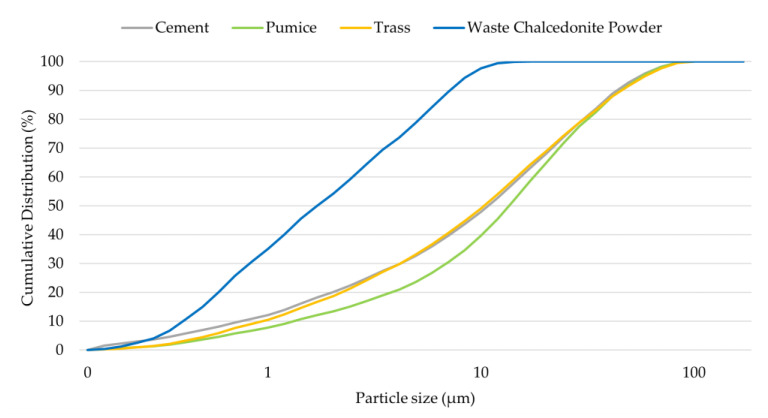
Particle size distributions of cement and additions.

The specific surface area of each raw material was tested by Blaine’s method [46] and was equal to 3686 cm^2^/g for cement, 8250 cm^2^/g for pumice, 6210 cm^2^/g for trass and 13,164 cm^2^/g for waste chalcedonite powder. The high specific surface areas of the particles of pumice, trass and waste chalcedonite powder had a significant effect on their pozzolanic activity. Table 2 represents the pozzolanic activity of pumice, trass and waste chalcedonite powder as tested by the Chappelle test [47]. In this method, the material is considered to be pozzolanically active when the amount of reacted Ca(OH)_2_ within the tested material after 24 h is at least 650 mg Ca(OH)_2_/1 g. As shown by the research performed, pumice and waste chalcedonite powder met the condition of pozzolanity. On the other hand, trass did not reach the required values even after 3 days of reaction and was evaluated as inactive. The low pozzolanic activity values of the trass were probably caused by the small specific surface area of its particles. The particle size distributions of the cement and all additives are shown in Figure 1. These were determined by Helos KR laser diffraction analyzer dry dispersion (Sympatec GmbH, Clausthal–Zellerfeld, Germany). Waste chalcedonite powder was characterized by the smallest grain size; conversely, pumice had the biggest grain size. The particle size distributions of cement and trass were similar. Cement and all raw materials had particle sizes in the range from 0 to 100 µm.

### 2.2. Composition of Pastes

In total, six pastes were designed, for which the percentage compositions of all binders can be found in Table 3. The compositions of pastes for individual tests are presented in Table 4, Table 5 and Table 6. The main binder in the tested pastes was cement, which was partially replaced by 30% pumice (CP), trass (CT), waste chalcedonite powder (CCH) or a combination of pumice or trass with waste chalcedonite powder in the amounts of 15% each (CPCH; CTCH). Reference cement paste (C) without additives was also prepared. 

### 2.3. Methods

**Figure 2:**
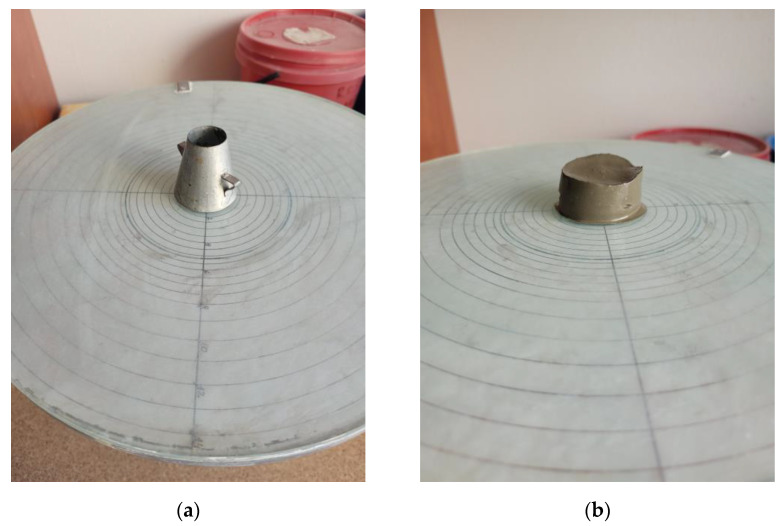
Consistency research: (**a**) view of the flow table and mini-cone form; (**b**) view of the sample.

**Figure 3:**
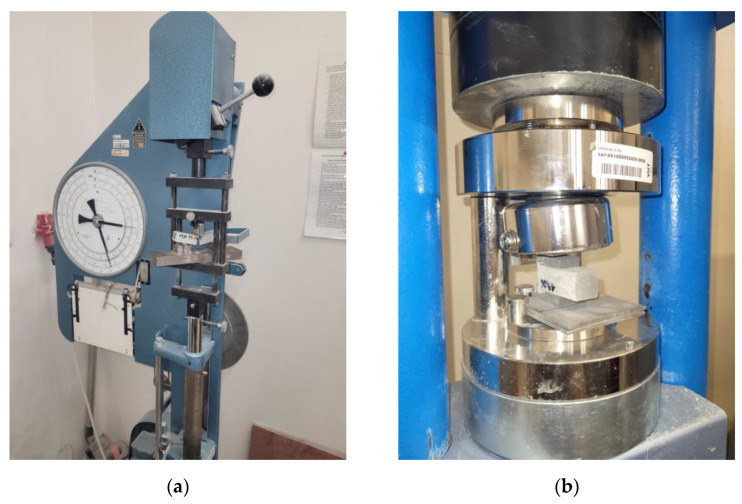
View of samples during research on mechanical properties: (**a**) flexural strength; (**b**) compressive strength.

**Figure 4:**
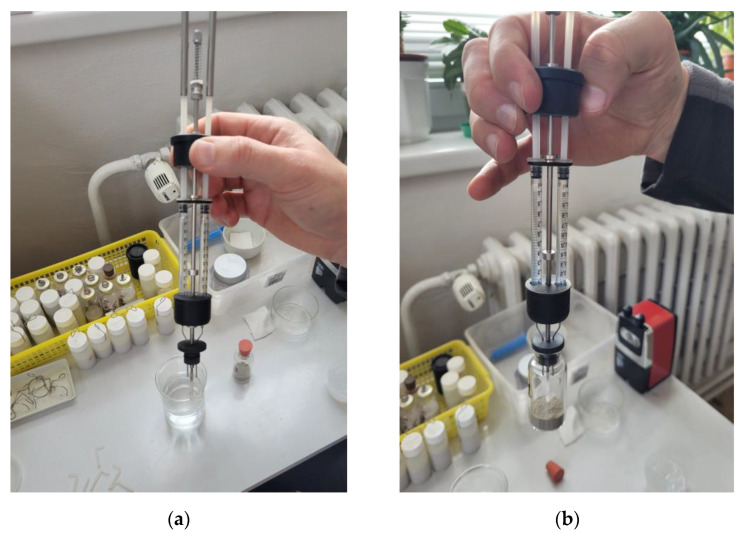
View of samples before calorimetric measurements: (**a**) view of ingredients; (**b**) view of the prepared sample.

- All ingredients were weighed with an accuracy of 0.1 g for most studies. Cement and additions for calorimetric measurements were weighed with an accuracy of 0.0001 g.
- Consistency was tested with a mini-slump cone test [48]. The research was performed on a flow table (Figure 2a). The mini–cone form was filled with paste and then the form was removed. Firstly, flow diameter was measured with a caliper four times. Secondly, the height (*H*) of the sample after flow was tested each time (Figure 2b). The dry ingredients were mixed with tap water manually. The mixing procedure was 1 min of mixing, then a 2 min break, then 1 min of mixing followed by 1 min break, then measurement.

- Rheological experiments were done using a Discovery HR–1 (TA Instruments, New Castle, DE, USA) accompanied by a DIN rotor. Flow curves were obtained 5 min and 30 min after adding water to the binder(s) (the first contact of water, cement and additives) at the temperature of 20 °C. The shear rate gradually changed from 1 s^−1^ to 100 s^−1^ and then from 100 s^−1^ to 1 s^−1^. The Herschel–Bulkley model [49] was used to calculate the following parameters: yield stress (*τ*_0_), consistency coefficient (*K*) (analogous to plastic viscosity) and fluidity index (*n*); thixotropy was calculated using the TRIOS software based on the hysteresis area between two flow curves. The dry ingredients were mixed with tap water. The mixing procedure involved 1 min of mixing manually then a 2 min break, followed by placing of the sample in the rheometer cylinder and then measurement.
- The paste mixtures for testing hardened materials were prepared in 20 mm × 20 mm × 100 mm moulds. Raw materials and water were mixed for 1 min manually and then the moulds were filled with paste. All of the samples were stored in plastic bags for 24 h to avoid water evaporation; afterwards, they were removed and placed in boxes on a grid above the water level. Mechanical properties tests were performed after 7 and 28 days of curing. Flexural strength (Figure 3a) was determined using a standard three–point bending test (the spacing between the supports was equal to 7 cm), and compressive strength (Figure 3b) was measured on the far edge of both residual pieces obtained from the flexural test. The compressed surface was a square with sides of 2 cm × 2 cm. The average of three measurements for each type of paste was taken as the final flexural strength and the average of six measurements for each type of paste was taken as the final compressive strength.

- Porosity, bulk density, apparent density and total pore area were measured with mercury intrusion porosimetry (MIP) using a PoreSizer 9310 (Micromeritics BV, Eindhoven, The Netherlands), generating a maximum pressure of 207 MPa and making it possible to evaluate a theoretical pore diameter of 0.006 μm. The measurements were performed on the cut part of the hardened paste, including both the interior and surface of the paste. Each paste was measured three times, and the results were averaged. MIP tests were performed on the samples remaining after mechanical properties research.
- Hydration processes were studied with calorimetric measurements using TAM Air 8–channel isothermal microcalorimeters (TA Instruments, New Castle, DE, USA). The changes in heat evolution for the first 7 days of the setting and hardening processes were monitored. The temperature of the calorimeter was constant and equal to 25 °C. Additionally, the degree of hydration was calculated for all samples, according to the pattern given in publication [50]. All dry ingredients were weighed and poured into a measuring box (small jar) (Figure 4a) and then the appropriate amount of water was measured (Figure 4b). The samples were seasoned in a calorimeter for 24 h. After this time, water was added to the dry ingredients, the mixture was mixed, and the measurements began.

## 3. Results and Discussion

### 3.1. Physical Properties

The results for the consistency of all tested pastes are presented in Table 7. 

Pastes containing additives had lower flow and a higher *H* parameter, mainly because of their smaller grain size and larger specific surface area versus cement. Mini-slump flow was reduced by about 33–37%. Pumice and trass additions decreased mini-slump flow by about 33%, waste chalcedonite powder by about 37%, a combination of pumice and waste chalcedonite powder by about 35%, and a combination of trass and waste chalcedonite powder by about 33%. The influence of 30% waste chalcedonite powder (CCH paste) on consistency was the most significant (due to it having the smallest grain size among all additives). The addition of waste chalcedonite powder in three-component pastes resulted in their slight thickening. The results of this research are consistent with the conclusions from publication [15], but opposite to the results from publication [33].

**Figure 5:**
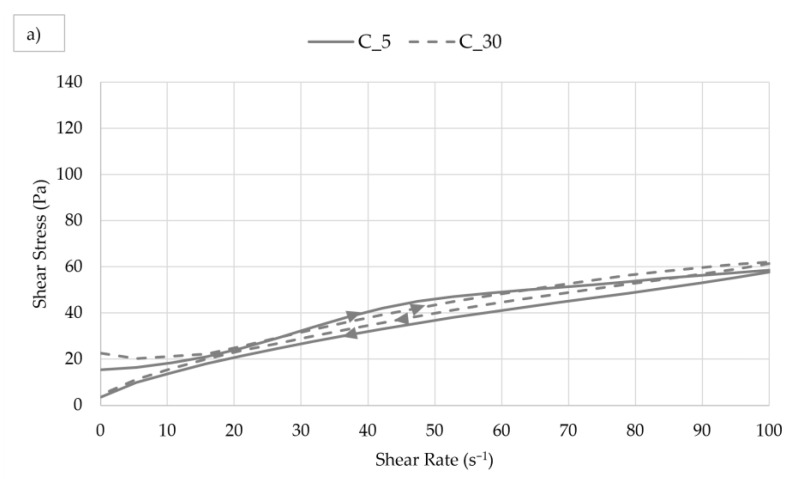

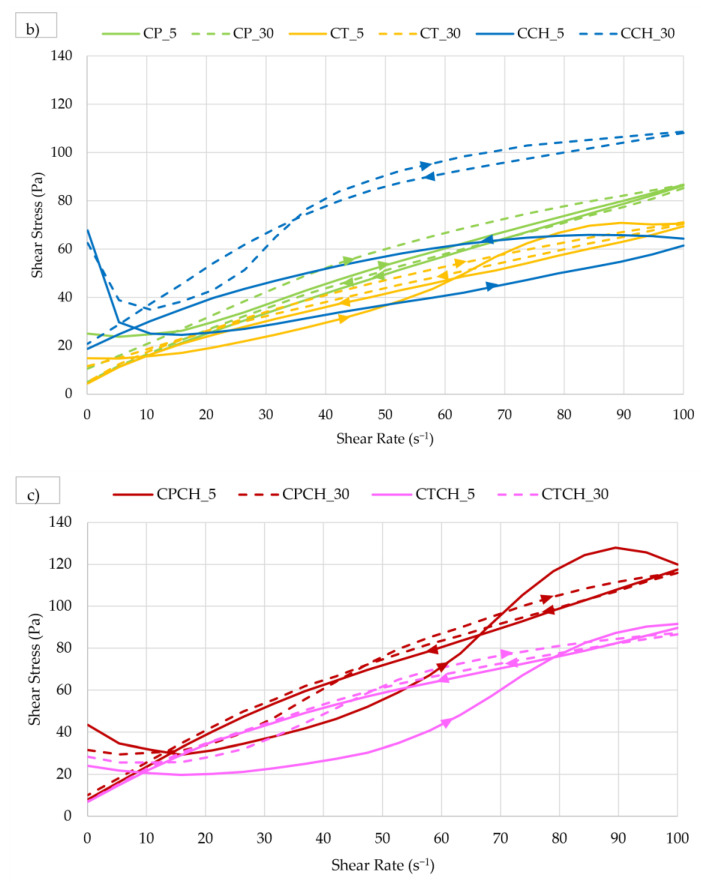
Flow curves for: (**a**) cement paste; (**b**) pastes with one additive; (**c**) pastes with a combination of two additives.

**Figure 6:**
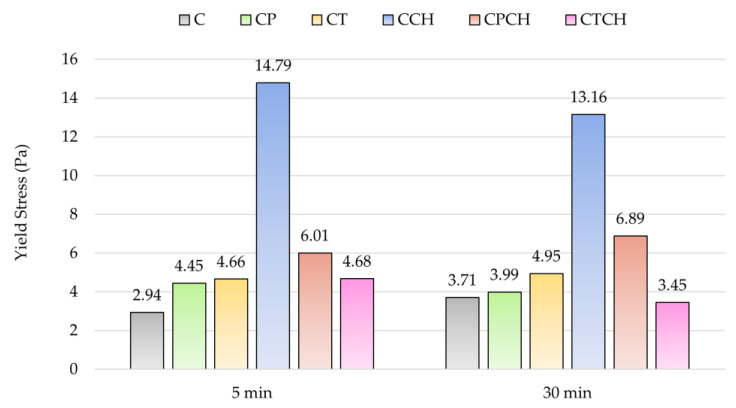
Results for the yield stress of pastes over time.

**Figure 7:**
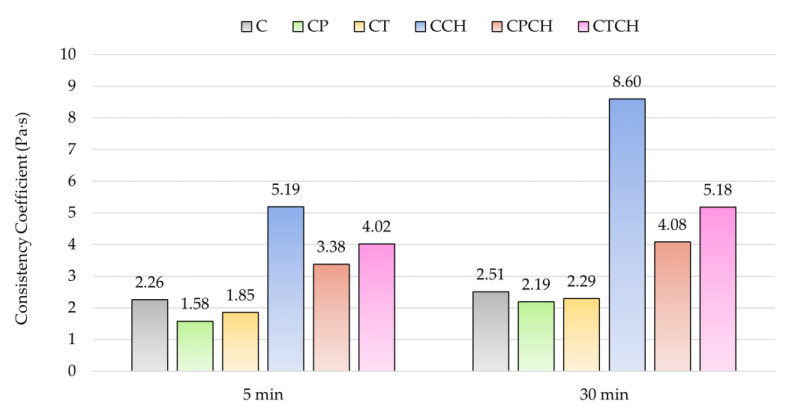
Results of consistency coefficient of pastes over time.

**Figure 8:**
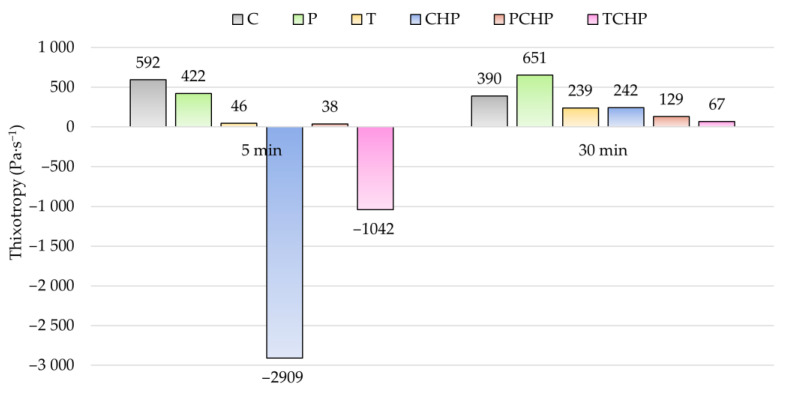
Results for the thixotropy of pastes over time.

The results concerning the rheological measurements of pastes are introduced in Table 8. The flow curves of cement paste and cement pastes with 30% additive(s) are presented in Figure 5. Results for yield stress, consistency coefficients and thixotropy measured after 5 and 30 min are shown in Figure 6, Figure 7 and Figure 8. 

A detailed look at the flow curves of the pastes shows that a denser structure of the pastes was formed over time, as the flow curves measured 30 min after mixing lie at higher shear stress values than the 5 min flow curves. The curves also indicate the thixotropic behavior of the pure cement paste, meaning that it thinned over time during mixing. Pastes with additives showed worse thixotropy (Table 8, Figure 8), and CCH and CTCH pastes were even rheopectic at the initial stage. This deterioration of flow properties during mixing was probably caused by increased absorption of water on the large surfaces of the additive particles. Over time, the thixotropic behavior of all pastes improved. The change in the composition of the cement pastes did not have a significant effect on the character of their behavior with increasing shear rate; all pastes were pseudoplastic (shear-thinning, *n* < 1). 

Analysis of rheological results showed that the majority of pastes with additive(s) were characterized by higher yield stress compared to the control sample (paste C), which is in agreement with the results of consistency measurement (lower flow values). On the other hand, the results for the consistency coefficient (plastic viscosity) were different. Pumice and trass negatively affected the *K* parameter compared to reference cement paste. Both waste chalcedonite powder and a combination of waste chalcedonite powder with pumice or trass positively affected the viscosity to higher values. Despite the smaller grain sizes of all three additives compared to cement, only waste chalcedonite powder increased the viscosity of the modified pastes. Waste chalcedonite powder acts as a thickening agent, which is mainly due to the large specific surface area of its particles. It is necessary to apply a higher shear stress to make such mixtures flow, and they flow less under the same conditions (they have higher viscosity). The incorporation of waste chalcedonite powder into cement pastes also increased the crystalline particle content, as chalcedonite consists of porous quartz grains with cryptocrystalline SiO_2_ [42], and mixtures with a higher crystalline content are more reluctant to flow. The individual crystals have to be arranged in the shear direction and require greater force to move, resulting in higher shear stress values. Waste chalcedonite powder affects the rheological properties of cement pastes very similarly to another natural pozzolan, spongilite [51]. 

The macro−structural properties of the tested pastes are presented in Table 9. The tested pastes were characterized by bulk densities in the range of 1.38–1.56 g/cm^3^, the highest being for the reference C sample. On the other hand, the apparent density ranged from 2.09 g/cm^3^ to 2.26 g/cm^3^, being highest for the C and CCH samples. The total pore areas for all materials varied. The reference sample (paste C) had a total pore area equal to 22.67 m^2^/g. This parameter of pastes with addition(s) was higher by 29–35% than for the reference sample, the highest value being for the sample with the addition of pumice. The porosity of the control paste C was equal to 31.12%, but this variable was in the range of 32.47–36.86% for the modified materials. Among all pastes with additives, the paste with waste chalcedonite powder had the highest porosity (36.86%). This may be caused by the nature of the stone powder. All additives significantly increased total pore area, but only slightly increased porosity. Interestingly, the material with the highest porosity (CCH) had a relatively low total pore area.

**Figure 9:**
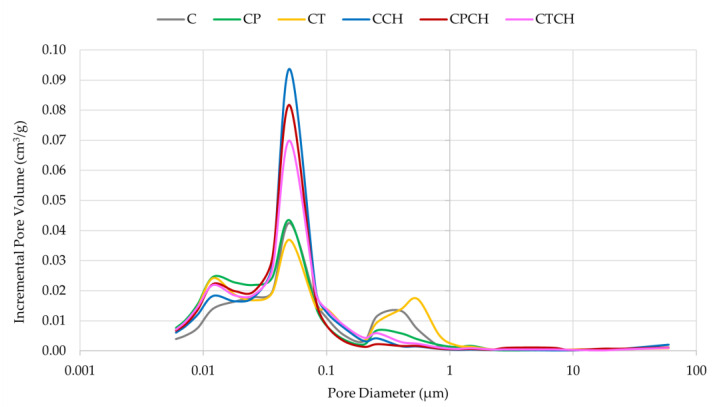
Pore size distributions in the tested pastes.

Incremental pore volume is shown graphically in Figure 9. This parameter varied depending on the type of addition(s). The addition of waste chalcedonite powder (in any case) increased the pore diameter in the range of 0.01–0.1 μm compared to the reference sample and pastes with pumice and trass. On the other hand, the change in pore diameter was the highest for cement paste and paste with trass, in the range of 0.01–1.0. Increasing the volume of larger pores at the expense of smaller pores resulted in a decrease in the bulk density and a reduction in the total pore area. Waste chalcedonite powder increased the size of smaller pores, but the addition of trass caused the pores to become larger in diameter.

### 3.2. Mechanical Parameters

**Figure 10:**
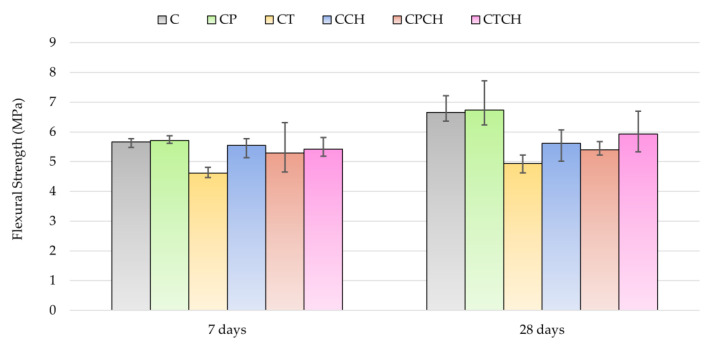
Results for the flexural strengths of pastes over time.

**Figure 11:**
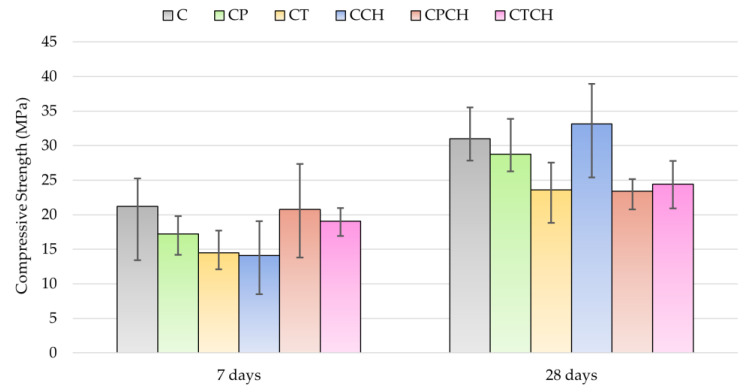
Results for the compressive strengths of pastes over time.

The results for flexural and compressive strength after 7 and 28 days are compared in Table 10 and Table 11 and Figure 10 and Figure 11. Additionally, changes in strength over time were calculated. Flexural strength measured after 7 and 28 days was the highest for samples with pumice. These results were similar to the results for the reference sample. The highest decrease in flexural strength after 28 days was recorded for the paste with trass (about 25.7% compared to the C sample). The compressive strength results were different. Cement paste had the highest compressive strength after 7 days. Pastes with a combination of two additives were characterized by the highest compressive strength (from modified pastes). The lowest compressive strength was noted in the case of the paste with waste chalcedonite powder, but its strength increased with time. The increase in strength between days 7 and 28 was equal to about 13%. Pumice and waste chalcedonite powder had slightly different effects when comparing compressive strength results after 7 days. Paste with pumice had a higher strength after 7 days than paste with stone powder and similar results to the reference sample. On the other hand, the result for compressive strength (after 28 days) was the highest for paste with waste chalcedonite powder. The pozzolanic activity of the waste chalcedonite powder was the highest among the three additives but required a longer time to become active. Partial replacement of the pumice with waste chalcedonite powder did not negatively affect the 28-day compressive strength of the pastes. In the case of pumice and waste chalcedonite powder, a paste containing both additives was characterized by a decrease of about 19% in strength compared to a sample containing only pumice.

### 3.3. Calorimetric Measurements

**Figure 12:**
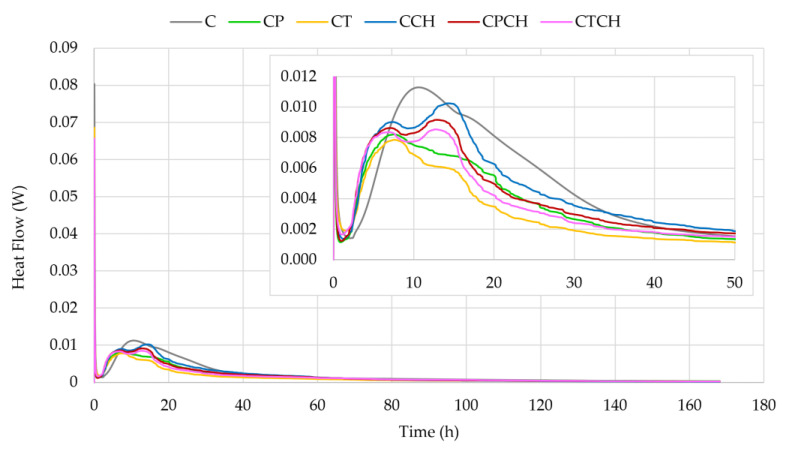
Heat flows of pastes over time.

**Figure 13:**
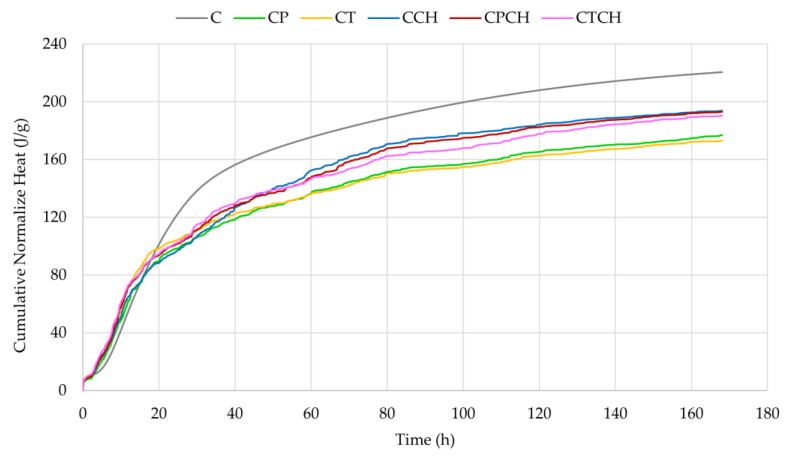
Cumulative normalized heat of pastes over time.

The heat of the hydration processes and the cumulative normalized heat of the tested pastes are presented in Figure 12 and Figure 13. The influences of pumice, trass and waste chalcedonite powder on the mentioned pastes were studied to note the mechanisms of setting and hardening processes. The results for the heat release rate indicate that the additives and their combinations affected the hydration processes of cement. The courses of the hydration curves varied depending on the type of additive(s), which is probably related to the pozzolanic activity of the raw materials. The second maximum (the main peak) was noted in both modified samples after about 6–7 h of hydration (earlier than in the case of the reference sample, cement paste). The initial signal is attributed to the dissolution and wetting of cement grains and also to the incipient formation of ettringite. The incorporation of additives led to a reduction of but an acceleration in heat flow. The induction time for all cement pastes was about 1.5 h. Based on the course of the curves (Figure 12), it can be concluded that the use of the additive(s) accelerates hydration processes, which is represented by the second peak being within the first 2–3 h of measurements (peak corresponding to C_3_S hydration). The extra-fine particles in the system increase the heterogeneous nucleation effect, and the dormant period is shorter. The third peak is associated with the point of solid gypsum exhaustion and the accelerated reactivity of the aluminate phase. The third peak appears as a small shoulder for plain cement (after 15 h) and progresses to a clearer peak with increasing waste chalcedonite powder content. The period of occurrence is accelerated for cement pastes with additives. Trass appears to have the least effect on aluminate hydration, followed by pumice. Tydlitát et. al. stated that the lower gypsum content and the increase in reactive aluminum content in blended cement pastes are behind the accentuation of heat development during the hydration of aluminate phases [52]. However, in the current research, it was found that the intensification of heat release in the same time range occurs in the case of the addition of waste chalcedonite powder, where no active aluminum is present. Therefore, the resulting paste contains a lower content of aluminates than pure cement paste. Similar results were obtained for cement pastes with 10% and 20% replacement of cement by silica fume, i.e., addition without active aluminum [53]. In contrast, cement pastes with pumice and trass, which contained a larger amount of Al_2_O_3_ than cement, did not show any significant changes in the hydration of aluminates compared to the reference paste. 

The cumulative normalized heat in the paste after 168 h is the highest in the reference sample (without additives). However, cement pastes with chalcedonite powder and a combination of waste chalcedonite powder with pumice or trass had higher cumulative heat (Figure 13 and Table 12) and degree of hydration (Table 13) values than cement paste with pumice and trass. According to accumulated heat−release data, the stimulation effect of additives occurred up to 15–17 h. Very similar results were obtained when natural zeolite or metakaolin was used as a pozzolanic additive to cement pastes [54,55].

## 4. Conclusions

The results of this research have shown that pumice, trass and waste chalcedonite powder can be components of blended Portland cements. Partial replacement of pumice, trass and waste chalcedonite powder in cement pastes has significant importance from an ecological and economical point of view. The use of such solutions is consistent with the principles of sustainable development. The properties of pastes with two- and three-component binders vary, depending on the types of additive(s).

1. All additives significantly reduced the flow of cement pastes. They are more water-demanding than cement. This is related to their larger specific surface area and the grain sizes of all additives compared to cement. From a practical point of view, this means that the consistency of mortars or concretes with these raw materials will be worse. Obtaining a flow similar to the reference sample would require an increase in the amount of water or the use of a plasticizer or superplasticizer.
2. Fresh cement pastes generally behave as Hershel–Bulkley fluids with very low yield stress. All the additives used had a thickening effect on the cement pastes, despite their different chemical and mineralogical compositions and specific surface areas. They increased the viscosity and yield stress and decreased the thixotropy of the pastes, which is consistent with the results of the consistency test.
3. Cement paste was characterized by the highest bulk density and apparent density. The use of additives increased the total pore area more than the porosity of the pastes. The increase in the porosity and total pore size of pastes with additives may be reflected in the greater vapor permeability of the materials.
4. The test results show that substitution of cement with 30% pumice, trass or waste chalcedonite powder induces a decrease in flexural and compressive strength after 7 days, but the best compressive strength after 28 days was obtained for the cement paste modified with 30% waste chalcedonite powder.
5. The use of the additive(s) accelerates the hydration processes of cement paste. The stimulation effect of additives occurs mainly at the early stages, up to 15 h, which may be reflected in the acceleration of the binding and hardening processes of these pastes. The total evolved heat of blended cement pastes is lower.
6. Replacing pumice or trass with 50% waste chalcedonite powder does not significantly deteriorate their parameters for consistency and porosity. Yield stress and consistency index are increased. Partial replacement of trass with chalcedonite powder resulted in a decrease in flexural strength, but the stone waste included in the pumice–chalcedonite pastes resulted in an increase in flexural strength after 7 and 28 days. The early compressive strength of paste with a three-component binder is higher than that of pastes modified with only one additive. Moreover, there is no significant deterioration in compressive strength after 28 days when part of the pumice or trass is replaced with waste chalcedonite powder. The use of three-component binders increases the cumulative amount of heat released in the paste and increases the degree of hydration (after 168 h) compared to pastes with pumice or trass.

## Figures and Tables

**Table 1:** Chemical composition of cement, pumice, trass and waste chalcedonite powder.

Components (%)	Cement (C)	Pumice (P)	Trass (T)	Waste Chalcedonite Powder (CH)
SiO_2_	18.33	54.27	50.08	99.01
Al_2_O_3_	4.71	20.50	17.61	0.84
Fe_2_O_3_	4.25	2.07	5.46	0.04
CaO	64.13	0.65	4.16	0.05
MgO	1.65	0.11	1.70	0.03
Na_2_O	0.05	9.20	3.61	0.05
K_2_O	0.59	5.62	4.67	0.04
SO_3_	2.68	0.07	0.05	0.00
TiO_2_	0.26	0.21	0.81	0.02
MnO	0.19	0.42	0.21	0.01
P_2_O_5_	0.16	0.07	0.34	0.03
L.O.I.	2.99	6.38	10.05	0.07

**Table 2:** Pozzolanic activity of the additives.

Pozzolanic Activity (mg Ca(OH)_2_/1 g)	Pumice	Trass	Waste Chalcedonite Powder
After 1 day	887	516	824
After 2 days	910	618	1201
After 3 days	958	641	1382

**Table 3:** Percentage compositions of all binders.

Component (wt. %)	C	CP	CT	CCH	CPCH	CTCH
Cement	100	70	70	70	70	70
Pumice	0	30	0	0	15	0
Trass	0	0	30	0	0	15
Waste Chalcedonite Powder	0	0	0	30	15	15

**Table 4:** Compositions of pastes for consistency and rheological research.

Component (g)	C	CP	CT	CCH	CPCH	CTCH
Cement	70	49	49	49	49	49
Pumice	0	21	0	0	10.5	0
Trass	0	0	21	0	0	10.5
Waste Chalcedonite Powder	0	0	0	21	10.5	10.5
Water	35	35	35	35	35	35

**Table 5:** Compositions of pastes for research on mechanical properties and MIP * research.

Component (g)	C	CP	CT	CCH	CPCH	CTCH
Cement	150	105	105	105	105	105
Pumice	0	45	0	0	22.5	0
Trass	0	0	45	0	0	22.5
Waste Chalcedonite Powder	0	0	0	45	22.5	22.5
Water	75	75	75	75	75	75

* MIP—mercury intrusion porosimetry method.

**Table 6:** Compositions of pastes for calorimetrical measurements.

Component (g)	C	CP	CT	CCH	CPCH	CTCH
Cement	4	2.8	2.8	2.8	2.8	2.8
Pumice	0	1.2	0	0	0.6	0
Trass	0	0	1.2	0	0	0.6
Waste Chalcedonite Powder	0	0	0	1.2	0.6	0.6
Water	2	2	2	2	2	2

**Table 7:** Consistency of all pastes.

Type of Paste	Mini–Slump Flow (mm)	*H* (mm)
C	61.3 ± 1.3	14.5
CP	41.0 ± 1.0	26.4
CT	41.2 ± 0.3	21.8
CCH	38.4 ± 0.9	28.3
CPCH	39.7 ± 0.5	26.4
CTCH	40.8 ± 0.1	24.4

**Table 8:** Rheological parameters of the pastes.

Type of Paste	Time after Mixing (min)	τ_0_ (Pa)	K (Pa·s)	n (−)	R^2^	Thixotropy (Pa·s^−1^)
C_5	5	2.94	2.26	0.69	0.99971	592
C_30	30	3.71	2.51	0.68	0.99977	390
CP_5	5	4.45	1.58	0.86	0.99998	422
CP_30	30	3.99	2.19	0.78	0.99957	651
CT_5	5	4.66	1.85	0.77	0.99912	46
CT_30	30	4.95	2.29	0.73	0.99951	239
CCH_5	5	14.79	5.19	0.68	0.99431	−2909
CCH_30	30	13.16	8.60	0.53	0.98294	242
CPCH_5	5	6.01	3.38	0.75	0.99908	38
CPCH_30	30	6.89	4.08	0.71	0.99883	129
CTCH_5	5	4.68	4.02	0.66	0.99858	−1042
CTCH_30	30	3.45	5.18	0.61	0.99660	67

**Table 9:** Parameters from MIP research (pastes after 28 days maturing).

Type of Paste	Bulk Density (g/cm^3^)	Apparent Density (g/cm^3^)	Total Pore Area (m^2^/g)	Porosity (%)
C	1.56	2.26	22.67	31.12
CP	1.46	2.16	35.08	32.47
CT	1.41	2.14	32.06	33.84
CCH	1.43	2.26	32.40	36.86
CPCH	1.38	2.09	34.59	33.86
CTCH	1.42	2.21	34.59	35.71

**Table 10:** Flexural strengths and changes in the flexural strengths of all pastes over time.

Type of Paste	Flexural Strength (MPa)		Changes in Flexural Strength Compared to C (%)	
	After 7 Days	After 28 Days	After 7 Days	After 28 Days
C	5.66	6.65	-	-
CP	5.71	6.74	+0.9	+1.4
CT	4.61	4.94	−18.5	−25.7
CCH	5.54	5.62	−2.12	−15.5
CPCH	5.29	5.40	−6.5	−18.8
CTCH	5.42	5.93	−4.2	−10.8

**Table 11:** Compressive strengths and changes in compressive strengths of all pastes over time.

Type of Paste	Compressive Strength (MPa)		Changes in Compressive Strength Compared to C (%)	
	After 7 Days	After 28 Days	After 7 Days	After 28 Days
C	21.19	30.99	−	−
CP	17.24	28.76	−18.6	−7.2
CT	14.48	23.60	−31.7	−23.8
CCH	14.12	33.14	−33.4	+6.9
CPCH	20.79	23.40	−1.9	−24.5
CTCH	19.08	24.42	−10.0	−21.2

**Table 12:** Cumulative normalized heat of all pastes over time.

Type of Paste	Cumulative Normalized Heat (J/g)		
	After 24 h	After 41 h	After 168 h
C	119.18	157.74	220.68
CP	98.06	120.41	176.94
CT	103.73	123.49	173.10
CCH	95.42	127.94	193.72
CPCH	100.65	128.94	193.10
CTCH	101.55	132.17	190.46

**Table 13:** Degree of hydration of tested pastes over time.

Type of Paste	Degree of Hydration (−)		
	After 24 h	After 41 h	After 168 h
C	54.01	71.48	100.00
CP	44.44	54.56	80.18
CT	47.00	55.96	78.44
CCH	43.24	57.98	87.78
CPCH	45.61	58.43	87.50
CTCH	46.02	59.89	86.31

## Data Availability

Data are contained within the article.
